# The Polymerase Activity of Mammalian DNA Pol ζ Is Specifically Required for Cell and Embryonic Viability

**DOI:** 10.1371/journal.pgen.1005759

**Published:** 2016-01-04

**Authors:** Sabine S. Lange, Junya Tomida, Karen S. Boulware, Sarita Bhetawal, Richard D. Wood

**Affiliations:** 1 Department of Epigenetics and Molecular Carcinogenesis, The University of Texas MD Anderson Cancer Center, Smithville, Texas, United States of America; 2 The Graduate School of Biomedical Sciences at Houston, Houston, Texas, United States of America; St Jude Children's Research Hospital, UNITED STATES

## Abstract

DNA polymerase ζ (pol ζ) is exceptionally important for maintaining genome stability. Inactivation of the *Rev3l* gene encoding the polymerase catalytic subunit causes a high frequency of chromosomal breaks, followed by lethality in mouse embryos and in primary cells. Yet it is not known whether the DNA polymerase activity of pol ζ is specifically essential, as the large REV3L protein also serves as a multiprotein scaffold for translesion DNA synthesis via multiple conserved structural domains. We report that *Rev3l* cDNA rescues the genomic instability and DNA damage sensitivity of *Rev3l*-null immortalized mouse fibroblast cell lines. A cDNA harboring mutations of conserved catalytic aspartate residues in the polymerase domain of *REV3L* could not rescue these phenotypes. To investigate the role of REV3L DNA polymerase activity *in vivo*, a *Rev3l* knock-in mouse was constructed with this polymerase-inactivating alteration. No homozygous mutant mice were produced, with lethality occurring during embryogenesis. Primary fibroblasts from mutant embryos showed growth defects, elevated DNA double-strand breaks and cisplatin sensitivity similar to *Rev3l*-null fibroblasts. We tested whether the severe *Rev3l*^-/-^ phenotypes could be rescued by deletion of DNA polymerase η, as has been reported with chicken DT40 cells. However, *Rev3l*^-/-^ *Polh*^-/-^ mice were inviable, and derived primary fibroblasts were as sensitive to DNA damage as *Rev3l*^-/-^ *Polh*^+/+^ fibroblasts. Therefore, the functions of REV3L in maintaining cell viability, embryonic viability and genomic stability are directly dependent on its polymerase activity, and cannot be ameliorated by an additional deletion of pol η. These results validate and encourage the approach of targeting the DNA polymerase activity of pol ζ to sensitize tumors to DNA damaging agents.

## Author Summary

Translesion synthesis allows DNA replication to occur in the presence of damaged DNA. This process is mediated by low-fidelity DNA polymerases (such as pol ζ or pol η) that maintain genomic stability. The action of these polymerases is crucial to limit cancer. In mice, complete deletion of DNA pol ζ leads to embryonic lethality, and conditional deletion enhances tumorigenesis. Pol ζ is a large protein with many domains that interact with other essential proteins and maintain the structural integrity of pol ζ. It is not known if the polymerase activity of pol ζ mediates its essential activities. Using a cell culture complementation system and *in vivo* knock-in mice, our work shows that pol ζ–mediated maintenance of genomic stability in the presence of DNA damage is absolutely dependent on its DNA polymerase activity. Others have demonstrated in chicken cells that co-deletion of pol ζ and pol η rescues the pol ζ-dependent phenotypes, but our work in mice and in mouse cell culture does not support that conclusion. These results demonstrate the physiological importance of pol ζ polymerase activity, and show that employing small-molecule inhibitors of the polymerase reaction is a valid strategy for sensitizing tumor cells to chemotherapeutic agents.

## Introduction

In eukaryotes, DNA polymerase ζ (pol ζ) is critical for the tolerance of many types of DNA replication blocks, by playing a central role in translesion DNA synthesis (TLS). Primary replicative DNA polymerases (pol δ or pol ε) are stalled when they encounter many types of template DNA adducts or DNA sequences forming stable secondary structures. Such stalled replication forks are prone to formation of a dangerous DNA double-strand break. The process of TLS helps avoid catastrophes by using a lower fidelity DNA polymerase (such as pol ζ or pol η), to incorporate nucleotides across from a lesion. TLS may occur either in S phase during primary DNA replication or in G2 phase during post-replication DNA synthesis. In yeast and in mammalian cells, pol ζ is important for this process, but it leads to endogenous and DNA damage-induced point mutations because of errors introduced during TLS [1–5]. Elimination of the pol ζ catalytic subunit *Rev3l* in mice leads to death during embryogenesis (reviewed in [6]). Primary cells in culture also cannot survive in the absence of *Rev3l*, because chromosomal DNA breaks quickly accumulate [7, 8]. Circumvention of damage-dependent checkpoints by SV40 large T antigen (TAg) immortalization of cells or by *Tp53* knockout allows *Rev3l*-deficient cell lines to grow, but the cells continue to display gross chromosomal instability and DNA damage sensitivity [8–10]. Mice conditionally deleting *Rev3l* in a fraction of hematopoietic cells or in basal skin keratinocytes are viable, but exhibit enhanced tumor incidence, as a consequence of the chromosomal instability of *Rev3l*-null cells [7, 11]. The hypersensitivity of REV3L-defective cells to some clinically-used DNA damaging agents indicates that REV3L is a possible target for enhancing the sensitivity of tumors to chemotherapeutic agents [12].

**Fig 1:**
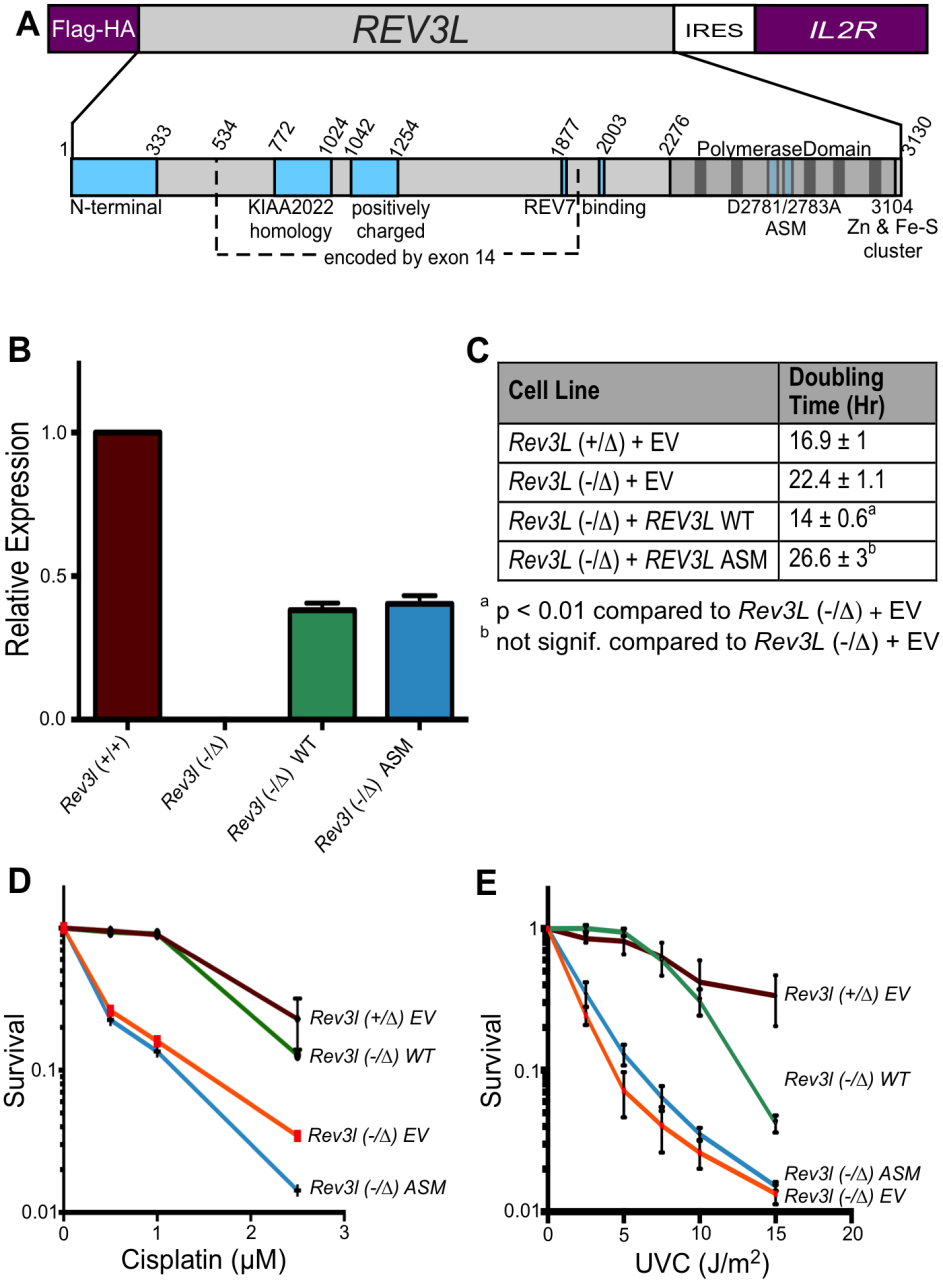
Expression of human *REV3L* complements *Rev3l*-deficient MEFs. (A) Top, the human *REV3L* gene was cloned into a pOZ vector for expression in mammalian cells with an N-terminal FLAG-HA epitope tag. The vector also expresses the interleukin 2 receptor (*IL2R*) gene via an internal ribosomal entry site (IRES). Below, domains in mammalian REV3L protein. Indicated here are the N-terminal domain, positively-charged domain, two REV7-binding domains, the KIAA2022 homology domain (see S1 Fig), and the C-terminal Fe-S cluster for interaction with other subunits. Vertical bars in the polymerase domain represent highly conserved motifs. The location of the D2781A/D2783A active site mutations (ASM) is shown. (B) Expression of *REV3L* in MEF cell lines. A set of primers and a Taqman probe were used that recognizes both human and mouse *Rev3l*, but does not amplify knockout transcript. Functional mouse *Rev3l* is expressed in *Rev3l*^+/+^ but not *Rev3l*^-/Δ^ cells. Wild-type or ASM recombinant *REV3L* mRNA was expressed in immortalized *Rev3l*^-/Δ^ MEFs at about half of the endogenous level. (C) Doubling time (in hr) of MEFs harboring empty vector (EV) *Rev3l*^+/Δ^, *Rev3l*^-/Δ^, and *Rev3l*^-/Δ^ MEFs expressing wild-type or ASM recombinant *REV3L*. (D) Survival of MEFs harboring empty vector (EV): *Rev3l*^+/Δ^ (maroon), *Rev3l*^-/Δ^ (orange); and *Rev3l*^-/Δ^ MEFs expressing wild-type (green) or ASM (blue) recombinant *REV3L*. ATP content was measured 48 hr after addition of cisplatin. (E) Survival of these cell lines 48 hr after ultraviolet C (UVC) radiation as measured by ATP content. Data represent mean ± SEM.

Although the consequences of pol ζ disruption are dramatic, it is not clear that these arise from a specific DNA polymerase defect. In mammalian cells, REV3L is a large protein (>3000 amino acids), with multiple functional domains. The DNA polymerase domain occupies only the last third of the protein (Fig 1A). The structural integrity of REV3L may be required in DNA processing complexes and for protein-protein interactions necessary to maintain cell viability and DNA integrity. Indeed, REV3L serves as a multi-DNA polymerase scaffold. The central region harbors two adjacent binding domains for REV7 (gene name *MAD2L2*). REV7 is necessary for pol ζ activity *in vitro* and serves an important function as a bridge protein for interaction with the REV1 protein [13–15]. REV1 in turn interacts with Y-family DNA polymerases that insert bases opposite sites of DNA damage and work in tandem with pol ζ [16–18]. REV7 also has other cellular functions in chromatin assembly and structure [19–21]. An N-terminal region of REV3 is conserved with yeast homologs [22]. At the C-terminus of REV3L [23], an Fe-S cluster is present that binds two other subunits of the pol ζ enzyme, POLD2 and POLD3. Both of these proteins also serve as subunits of the replicative DNA polymerase δ [23–26]. More recently, a conserved positively charged domain in the central region has been recognized as necessary for the efficient polymerase function of the recombinant protein [24]. Another domain in the central region has strong homology to the *KIAA2022* gene (S1 Fig).

A provocative hypothesis has been put forward to explain the severe genotoxic effects of *Rev3l* deletion [27]. It was suggested that these are the consequence of the function of a second DNA polymerase, pol η (gene *Polh*). As in mammalian cells, chicken DT40 cells with a disruption of pol ζ exhibit growth defects, chromosomal aberrations and DNA damage sensitivity [27]. Remarkably, it was reported that co-disruption of *Polh* and *Rev3l* corrects all of these phenotypes in DT40. The suggested interpretation was that pol η and pol ζ always work together in bypass of DNA damage, and that a toxic intermediate is formed by pol η that cannot be resolved in the absence of pol ζ. It is clearly important to determine, in mammalian cells, whether the genome instability caused by pol ζ disruption is dependent on pol η.

Here we describe experiments with knockout cells and a specific knock-in mouse model to test whether the catalytic activity of pol ζ is responsible for the phenotypes observed in pol ζ knockout mutants. We describe complementation of *Rev3l*-deficient mouse embryonic fibroblasts (MEFs) by expression of full-length human wild-type *REV3L*, and show that DNA polymerase-defective mutant REV3L cDNA is unable to complement cell survival or increased levels of DNA breaks. Using a *Rev3l* polymerase-dead knock-in mouse model, we show that specific disruption of the polymerase activity prevents the completion of embryogenesis. Finally, we tested whether pol ζ defects can be rescued by ablation of pol η function.

## Results

### Expression of *REV3L* cDNA in mouse embryonic fibroblasts rescues slow growth

*Rev3l* deletion in mouse cell lines is associated with an elevated baseline level of DNA breaks and an increased sensitivity to DNA damaging agents such as cisplatin and UV radiation [3, 8–10]. We wanted to test definitively whether these phenotypes are caused by the disruption of *Rev3l*. A pOZ expression vector harboring an IL2R selectable marker (Fig 1A) [28] was used to express human *REV3L* cDNA in *Rev3l*-deficient MEFs [8]. Cells were selected for IL2R expression by repeated cycles of magnetic bead sorting and clonal populations were isolated. The integrity of the expression vector was confirmed by PCR-based detection, and cells were assayed for expression of *REV3L* mRNA by real-time RT-PCR. Human *REV3L* was expressed in the *Rev3l*-deficient MEFs at about one-half of the normal endogenous level (Fig 1B). Mouse cells expressing one or two alleles of *Rev3l* have indistinguishable low levels of spontaneous senescence, apoptosis, and chromosome aberrations [8] and there is no haploinsufficiency apparent regarding embryonic or adult viability in mice [7].

We expressed both wild-type *REV3L (*WT*)*, as well as *REV3L* with a dual point mutation (ASM: D2781A; D2783A) in residues essential for divalent metal binding in conserved DNA polymerase motif I. Equivalent changes in all other tested DNA polymerases inactivate Mg^2+^ coordination in the active site, and eliminate enzymatic activity [29, 30].

We tested the growth of *Rev3l*-proficient and deficient cells expressing an empty vector (EV), as well as deficient cells expressing WT and ASM *REV3L* cDNA. *Rev3l*-deficient cells experienced S-phase associated delay and mitotic failure, leading to a population doubling time that was longer than *Rev3l*-proficient populations [8]. *REV3L* re-expression in the deficient cell lines significantly decreased their doubling time to a level similar to *Rev3l*^-/+^ cells, whereas expression of the polymerase-inactive mutant had no effect (Fig 1C).

### Rescue of DNA damage sensitivity and DNA breaks by *REV3L* expression

Deletion of *Rev3l* causes sensitivity to DNA damaging agents [8–10]. To determine whether *REV3L* expression could rescue this phenotype, cells were exposed to cisplatin or UVC radiation and cell survival was measured. *Rev3l*-deficient cells displayed the expected sensitivity to these damaging agents when compared to the *Rev3l*-proficient cells (Fig 1D and 1E). Assays were repeated with multiple clones for each genotype. Expression of wild-type *REV3L* rescued the sensitivity to all three DNA damaging agents, but expression of ASM *REV3L* did not.

**Fig 2:**
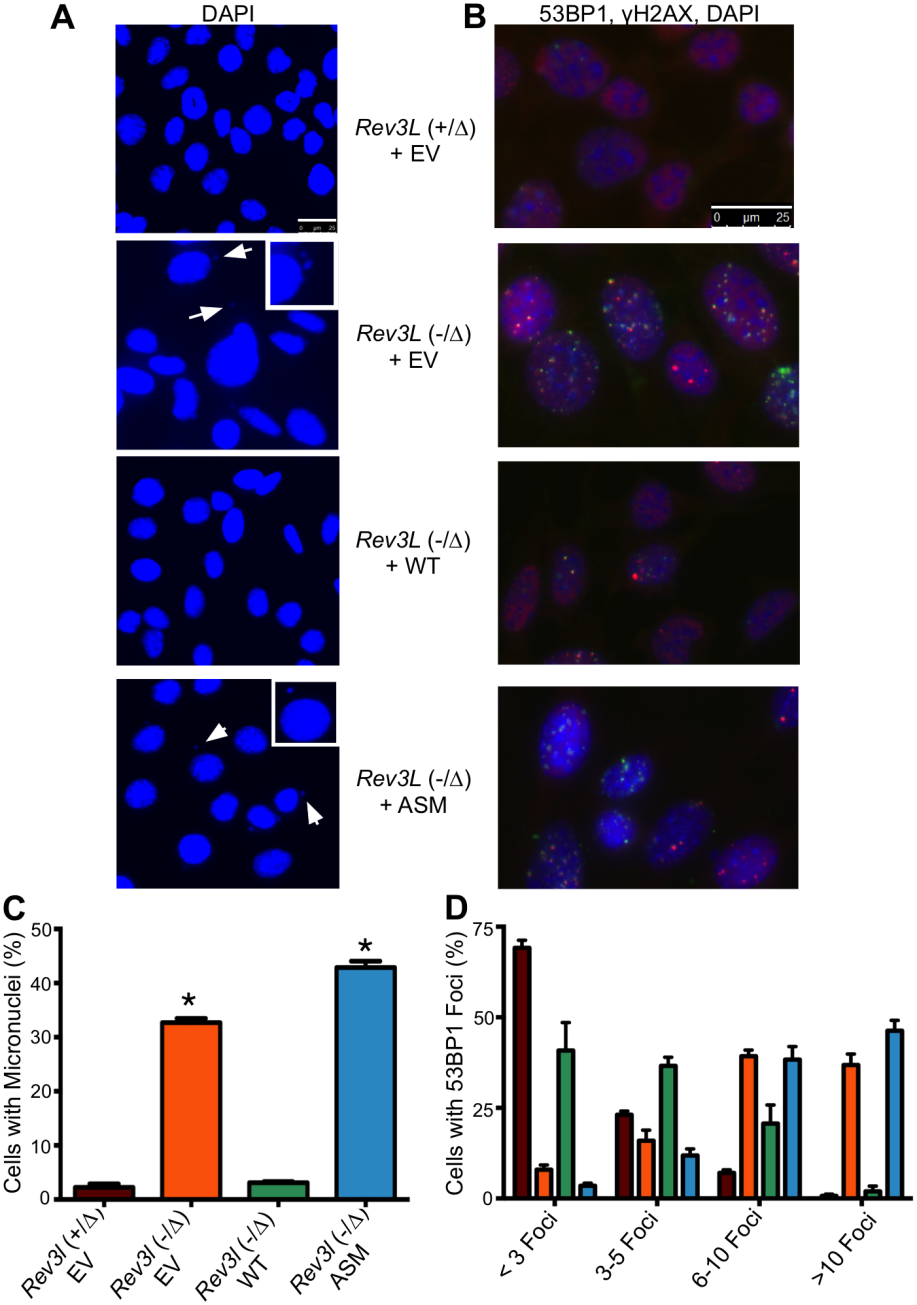
Spontaneous DNA double-strand break formation is reduced in *Rev3l* complemented MEFs. (A) DAPI staining of empty vector (EV)-expressing *Rev3l*^+/Δ^, *Rev3l*^-/Δ^, as well as *Rev3l*^-/Δ^ MEFs expressing wild-type or ASM recombinant *REV3L*; arrows indicate micronuclei, with an enlarged example in the inset. (B) Merged immunofluorescence staining of the same MEFs as in (A) with DAPI (blue), 53BP1 (red) and γ-H2AX (green); foci indicate areas of DNA double-strand breaks. (C) Quantification of percent of nuclei that have associated micronuclei. (D) Quantification of cells with fewer than 3, 3 to 5, 6 to 10 or greater than 10 53BP1 foci (as measured using CellProfiler). The bars are color-coded exactly as in Part C to indicate the genotype of the MEFs (*) p < 0.01. Data represent mean ± SEM.

*Rev3l*-deficient cells manifest an increased formation of DNA breaks in the absence of exogenous DNA damage. We measured a 10 to 20-fold increase in cellular micronuclei (Fig 2A and 2C) in *Rev3l*-defective cells, with 30–40% of all cells displaying micronuclei. The *Rev3l* defect was also accompanied by an increased frequency of DNA breaks as quantified by 53BP1 foci per cell, with a pronounced shift in distribution towards larger numbers of foci per cell (Fig 2B and 2D). Expression of wild-type *REV3L* in *Rev3l*-deficient MEFs rescued both of these phenotypes, but expression of ASM *REV3L* did not. The frequency of sister chromatid exchange (SCE) was not decreased in *Rev3l*-deficient cells (Table 1), indicating that this mitotic recombination event is not impaired by a *REV3L* defect. These experiments demonstrate that sensitivity to DNA damaging agents and the presence of DNA breaks in *Rev3l*-deficient cells is caused by the absence of the REV3L protein, and REV3L polymerase activity is required for prevention of these phenotypes.

**Table 1:** Sister Chromatid Exchange (SCE) frequency in immortalized MEF cell lines.

Cell line	*Rev3l* status	% Av SCE/chrom ± s.d.	Av no. chrom ± s.d.
*Rev3l*^+/Δ^ *TAg*	+/-	15.9 ± 7.9	72 ± 15
*Rev3l*^-/Δ^ *TAg*	-/-	27.3 ± 9.3	105 ± 29
*Rev3l*^+/+^ *Tp53*^-/-^	+/+	15.5 ± 6.2	80.9 ± 7
*Rev3l*^+/-^ *Tp53*^-/-^	+/-	23.9 ± 9.0	51.7 ± 11

SCE frequencies are given as the average number per chromosome (total SCE observed / total chromsomes counted). For each cell line, 30–35 metaphases were scored. All cell lines were polyploid, as is commonly observed following immortalization. One MEF cell line pair was derived from p53-defective embryos and were described by Wittschieben *et al*. [9]; these are the *Rev3l*^+/+^ *Tp53*^-/-^ B2 cell line and the *Rev3l*^+/-^ *Tp53*^-/-^ B4-9 cell line. The second MEF cell line pair was derived by T-antigen immortalization of primary cells as described [8], these were *Rev3l*^+/Δ^ 1(+)cl2 TG1Het1 and *Rev3l*^-/Δ^ 5(-)cl7 TG2Het5. No reduction of SCE frequency per chromosome was found in *Rev3l*-defective cells.

We also investigated two reported human REV3L knockout lines designated 332 and 504, derived from the Burkitt lymphoma cell line BL2 [31]. However, *Rev3l* mRNA is still transcribed in the 332 and 504 subclones, the subclones were no more sensitive to cisplatin than the parental BL2, there was no significant increase in spontaneous double-strand break incidence in the subclones, and no complementation of the mild UV sensitivity was observed with Rev3L cDNA (S2 Fig). These results and uncertainties regarding the targeting strategy (S3 Fig) indicate that the BL2 subclones may not be pol ζ defective.

### Specific inactivation of *Rev3l* DNA polymerase activity causes embryonic lethality

**Fig 3:**
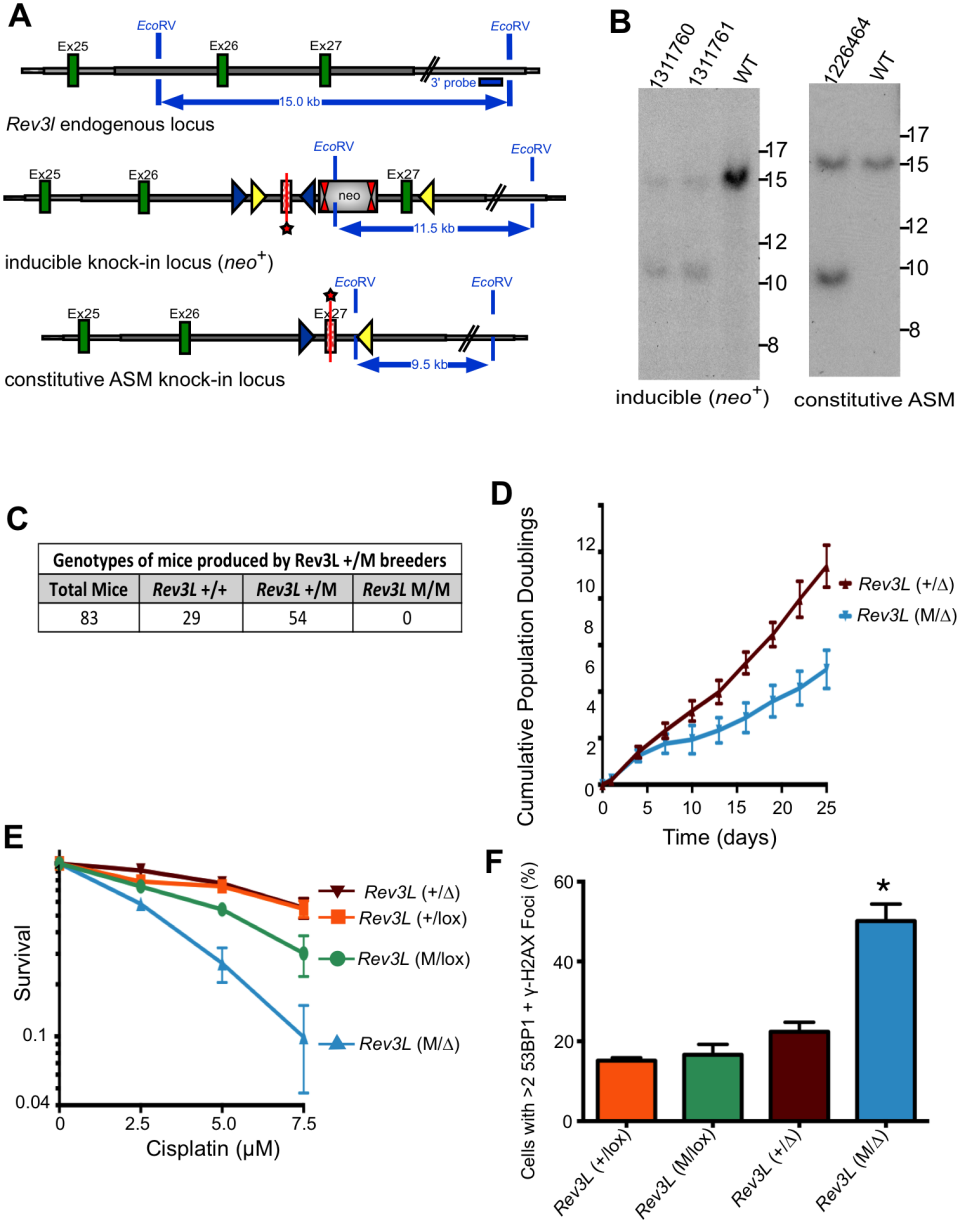
Knock-in mice and MEFs expressing active site mutant *Rev3l* have knockout phenotypes. (A) Diagram of the mouse *Rev3l* ASM knock-in allele, with the wild-type (WT) locus shown at the top. Green rectangles indicate *Rev3l* coding sequences and the gray line represents chromosomal sequence. In the middle diagram, FRT sites are represented by double red triangles, loxP sites by blue triangles and lox511 sites by yellow triangles. The targeted exon 27 (starred) carries D2773A and D2775A point mutations and is inserted in an inverted orientation between wild-type exons 26 and 27. Splicing donor and acceptor sites flanking wild-type and ASM exon 27 are kept intact. The knock-in was produced by a Cre-dependent genetic switch. First, the neomycin positive selection cassette (neo) was excised by breeding with C57BL/6 Flp deleter mice. A subsequent cross with Cre-expressing mice led to excision of the wild-type exon 27 and inversion of ASM mutant exon 27 into the functional orientation. In the constitutive ASM knock-in locus shown in the lower diagram, the D2773A/D2775A *Rev3l* gene is expressed under the control of the endogenous *Rev3l* promoter and wild-type *Rev3l* exon 27 is absent from the locus. Heterozygous ASM knock-in mice (*Rev3L*^+/M^) were then used for breeding. (B) Example of Southern blot analysis of (left) the inducible knock-in locus (*neo*^+^) and (right) the constitutive ASM knock-in locus. Genomic DNA of the tested animals was compared with C57BL/6 wild-type genomic DNA (WT). *Eco*RV digested DNA was blotted on a nylon membrane and hybridized with the external 3’ probe with the position shown at the top of part A. Restriction fragments of 15 kb, 11.5 kb and 9.5 kb were observed for the wild-type, inducible knock-in locus (*neo*^+^) and constitutive ASM knock-in locus, respectively. Genomic DNA was further analyzed extensively and confirmed by specific PCR assays and complete DNA sequencing as described in the Materials and Methods. (C) Genotypes of mouse pups produced by breeding parental *Rev3l*^+/M^ mice. (D) Growth of *Rev3l*^+/Δ^ and *Rev3l*^M/Δ^ cells. These cells were produced by addition of AdCre to *Rev3l*^M/lox^ or *Rev3l*^+/lox^ MEFs, deleting the floxed allele of *Rev3l*. (E) Survival of *Rev3l*^+/lox^, *Rev3l*^M/lox^, *Rev3l*^+/Δ^ and *Rev3l*^M/Δ^ primary MEFs 120 hr after addition of cisplatin, as measured by ATP content. (F) The MEFs as in (E) were stained with DAPI, and for 53BP1 and γ-H2AX by immunofluorescence as in Fig 2 to detect foci of DNA double-strand breaks. The quantification shows the percentage of cells with >2 53BP1 and γ-H2AX foci in *Rev3l*^+/lox^, *Rev3l*^M/lox^, *Rev3l*^+/Δ^ and *Rev3l*^M/Δ^ primary MEFs 9 days after AdCre treatment. (*) p < 0.01. Data represent mean ± SEM.

To determine the *in vivo* consequence of specifically inactivating the DNA polymerase function of *Rev3l*, a genetically engineered mouse was constructed to express an ASM knock-in allele from the endogenous promoter (Fig 3A). Variant lox sites [32] were used to control knock-in of the *Rev3l* allele. The mice were crossed to CMV-Cre, producing a constitutive ASM allele (abbreviated the “M” allele for the mice here), in a pure C57BL/6J background. All steps of genomic engineering were extensively monitored by Southern blotting analysis (Fig 3B), PCR analysis and DNA sequencing.

Heterozygote mutant *Rev3l*^*+/M*^ mice were viable and fertile, demonstrating that the mutant allele does not have dominant-negative activity affecting viability. Heterozygous mutant *Rev3l*^+/M^ mice were bred and pups genotyped. No homozygous mutant animals were identified at weaning (Fig 3C). In addition, 48 embryos from 6 pregnancies were isolated between 8.5 and 10.5 dpc. *Rev3l*^*M/M*^ embryos were rare at the earlier timepoints, and by 10.5 dpc only a few very small *Rev3l*^*M/M*^ embryos were identifiable. The severely impaired development of homozygous *Rev3l* ASM embryos mirrors the lethality of the *Rev3l* null allele on a C57BL/6 background [33].

### Growth defects and accumulation of DNA strand breaks in *Rev3l* ASM cell lines

Due to the early embryonic lethality in *Rev3l*^M/M^ embryos, we were never successful in deriving MEFs from them. To circumvent this problem we crossed *Rev3l*^+/M^ mice with *Rev3l*^-/lox^ mice. This mating produced embryos for derivation of viable *Rev3l*^M/lox^ MEFs. The floxed (lox) allele of *Rev3l* is functional, but becomes a knockout allele (termed the Δ allele) after action of the Cre recombinase. We expressed Cre recombinase in the cells to yield *Rev3l*^M/Δ^ MEFs. The mice also harbored the mT/mG transgene to monitor Cre activity. This mT/mG transgene constitutively expresses red fluorescent protein (RFP). When Cre is active, the RFP gene is removed and green fluorescent protein (GFP) is expressed [34]. This allows GFP to be used for flow sorting and as a marker of cells in which Cre recombinase has been expressed.

**Fig 4:**
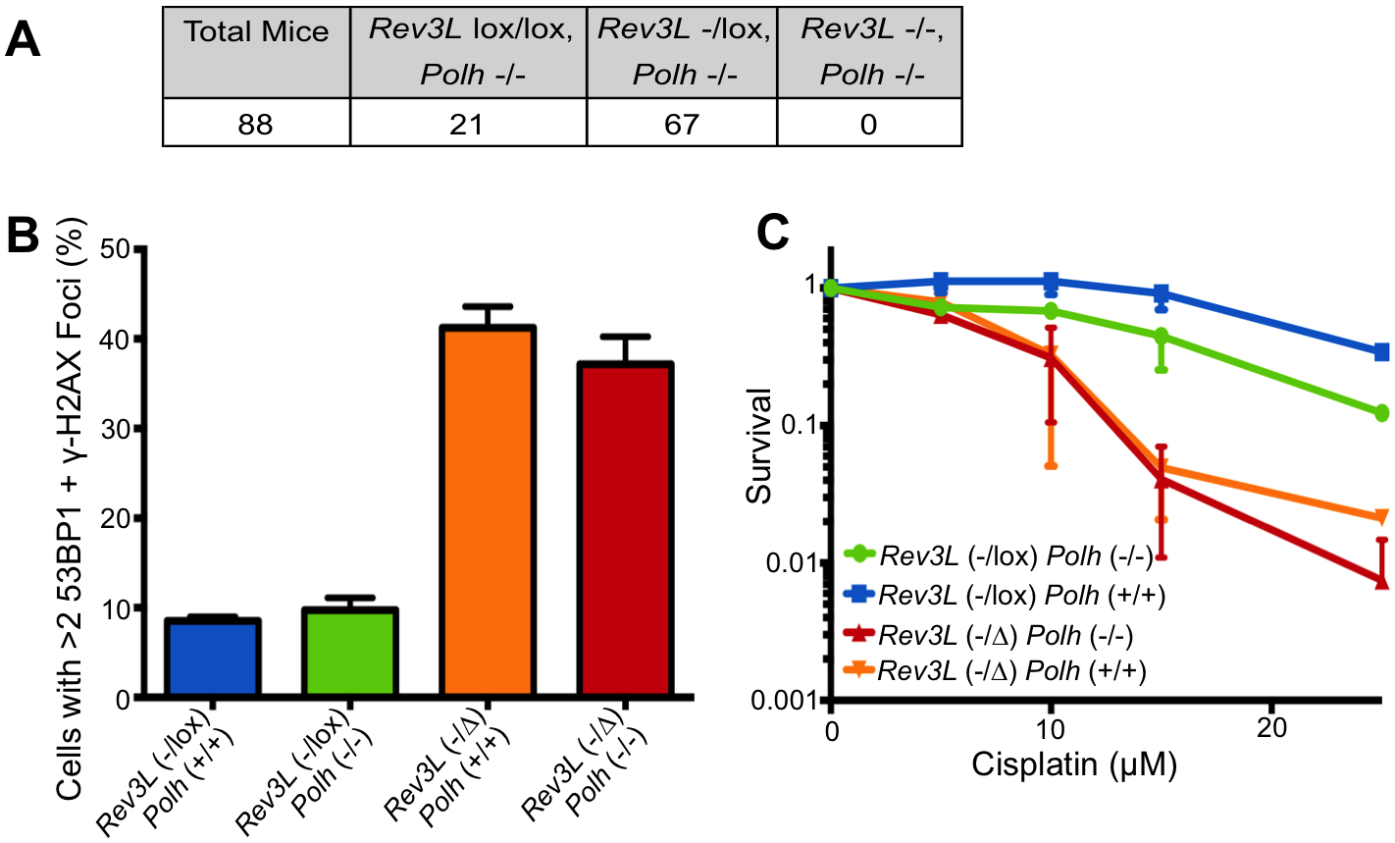
Deletion of *Polh* does not ameliorate phenotypes caused by knockout of *Rev3l*. (A) Genotypes of mouse pups produced by breeding parental *Rev3l*^-/lox^ *Polh*^-/^—mice. (B) MEFs with the indicated genotypes were stained with DAPI, and for 53BP1 and γ-H2AX by immunofluorescence as in Fig 2 to detect foci of DNA double-strand breaks. The quantification shows the percentage of cells with >2 53BP1 and γ-H2AX foci in *Rev3l*^-/lox^ *Polh*^+/+^, *Rev3l*^-/lox^ *Polh*^-/-^, *Rev3l*^-/Δ^ *Polh*^+/+^ and *Rev3l*^-/Δ^ *Polh*^-/-^ MEFs 9 days after AdCre treatment. (C) Survival of primary MEFs as in part B, 120 hr after addition of cisplatin, as measured by ATP content. For panels B& C, Data represent mean ± SEM.

Cre was introduced via an adenovirus vector into primary MEFs [8] to compare *Rev3l*^M/Δ^ MEFs with *Rev3l*^*M/+*^ MEFs (retaining a wild-type allele of *Rev3l*). We measured cell growth, cisplatin sensitivity and DNA double-strand breaks in GFP-positive cells. ASM MEFs had a growth defect compared to wild-type allele-containing MEFs (Fig 3D) and eventually failed to thrive. ASM MEFs were hypersensitive to cisplatin, compared to control MEFs (Fig 3E). Additionally, there was a two to three-fold increase in the number of ASM MEFs containing 53BP1 and γ-H2AX foci (a measure of DNA breaks) compared to controls at 9 days after Cre recombinase expression (Fig 3F). These phenotypes are similar to those seen in *Rev3l* null primary MEFs [8] (and compare Figs 3F and 4B). This result demonstrates that the DNA polymerase activity of REV3L is specifically required to allow for cell proliferation, to protect genome stability and to moderate cisplatin sensitivity.

### Deletion of DNA polymerase eta does not rescue *Rev3l*-deficient phenotypes in mice

We wanted to determine in mammalian cells whether the DNA damage sensitivity and genome instability caused by pol ζ disruption is dependent on pol η, as has been reported for the DT40 cell line [27]. We crossed parental mice with the genotypes *Rev3l*^*-/lox*^ *Polh*^-/-^, and investigated the genotypes of the pups. In the *Polh*^-/-^ background, no *Rev3l*^-/-^ mice were born (Fig 4A), consistent with the complete lethality of the *Rev3l*^-/-^ genotype in a *Polh*^+/+^ background [6]. We attempted to produce *Rev3l*^-/-^ *Polh*^-/-^ MEFs from mouse embryos, but were unable to obtain sufficient material to produce viable MEFs because of the early death during embryogenesis. Instead we derived primary MEFs from viable *Rev3l*^*-/lox*^ *Polh*^-/-^ embryos. Following introduction of Cre via an adenovirus, *Rev3l*^-/Δ^ *Polh*^-/-^ cells were produced. These *Rev3l-*defective primary MEFs had an elevated level of DNA breaks that was indistinguishable from *Rev3l*^-/Δ^ *Polh*^+/+^ cells (Fig 4B). Consistent with published results [35] the pol η defect in *Rev3l*^-/lox^ *Polh*^-/-^ MEFs conferred enhanced sensitivity to cisplatin (by comparison with *Rev3l*^-/lox^ *Polh*^+/+^ MEFs) (Fig 4C). A *Rev3l* defect independently enhanced cisplatin sensitivity, and the sensitivity of the *Rev3l*^-/Δ^ *Polh*^-/-^ and the *Rev3l*^-/Δ^ *Polh*^+/+^ MEFs was similar. Therefore, deletion of pol η does not rescue the cell and organismal defects caused by loss of pol ζ, showing that the absence of pol ζ does not create a pol η-dependent toxic intermediate in mouse cells.

## Discussion

### The polymerase activity of pol ζ is essential for embryonic development and for limiting genome damage

A major objective of this study was to determine whether the catalytic activity of pol ζ is responsible for the severe consequences observed in pol ζ mutant mouse cells. These include hypersensitivity to DNA damaging agents, a greatly increased generation of double-strand breaks in unchallenged cells, a slower growth rate, and a required role for pol ζ in embryonic viability. The impetus for this question is the existence of numerous other functional domains within the catalytic subunit of REV3L. These include a conserved N-terminal domain, two REV7 binding domains [14, 19], and a C-terminal Fe-S cluster that interacts with the POLD2 subunit and is necessary for *in vitro* activity. In addition, the central region contains a conserved positively charged domain [24] that likely promotes protein-protein and protein-DNA interactions, and a KIAA2022 homology domain, described in detail here for the first time (S1 Fig). The presence of all of these domains introduces the possibility that the essential functions of REV3L could be structural, rather than directly related to the DNA polymerase activity itself. A catalytically deficient but otherwise intact REV3L may have been able to specifically interact with protein partners and DNA substrates, allowing viability of cells and mice.

There is ample precedent for such a situation. One example is the mammalian *ERCC2/XPD* gene. Complete disruption of *XPD* is incompatible with viability [36]. However, an amino acid substitution that inactivates the catalytic helicase activity of XPD specifically compromises nucleotide excision repair capacity, but allows cellular viability [37]. This is because the presence of XPD as a subunit of transcription factor TFIIH is necessary for the integrity of that complex, even though XPD activity itself is unnecessary for transcription [38, 39]. Another example is provided by the REV1 protein. REV1 has a DNA polymerase domain that can catalyze dCMP incorporation in DNA. Cells lacking REV1 are hypersensitive to UV radiation, but this DNA damage tolerance activity does not require the polymerase catalytic domain of REV1. Instead, the damage tolerance activity is conferred by a protein-protein interaction domain at the C-terminus of REV1 that interacts with REV7 in pol ζ and with Y family DNA polymerases [40]. Recently, a non-catalytic role has been reported for human DNA pol κ in protection against oxidative stresses [41].

Here, we analyzed the consequence of a homozygous mutation of the *Rev3l* DNA polymerase active site. No viable homozygous mice were produced, and the corresponding embryos died early in embryogenesis, as with a complete knockout allele. To investigate cell-autonomous consequences of the specific polymerase alteration, we derived primary MEFs that carried one null *Rev3l* allele, and one active site mutant allele. The growth defects, DNA break formation and cisplatin sensitivity of these cells were similar to cells harboring two null alleles [8]. These results show that the DNA polymerase activity of REV3L is essential for all functions so far measured in mice and in cells.

### Rescue of phenotypes by expression of the wild-type *REV3L* gene

Loss of *Rev3l* causes chromosomal instability in cells. This complicates studies of the consequences of *Rev3l* deficiency, as genomic alterations may accumulate during each cell cycle and lead to new phenotypes. A rigorous way to determine which phenotypes are directly caused by *Rev3l* loss is to complement the cells by expression of *Rev3l* cDNA. Here we utilized a complementation system for REV3L in mammalian cells, allowing definitive testing of whether phenotypes seen in *Rev3l*-deleted cells are due to *Rev3l*-deletion [19, 42]. Our results with specific mutant cDNAs establish that the polymerase activity of REV3L is specifically essential for preserving genome integrity and protecting against DNA damage. It is of course possible that other domains within REV3L also have critical functions for viability or genome integrity, and this complementation system will allow investigation of that possibility. For example, we recently demonstrated that the REV7-binding domains of REV3L are essential for pol ζ function [19].

We also attempted complementation of *Rev3l*-deficient phenotypes using human BL2 cell lines, reported to carry disruptions of *REV3L* [31]. It is notable that there were no major differences in phenotypes between the wild-type BL2 cells and the nominal 332 and 504 *REV3L* mutants. In contrast to the marked phenotypes found with *Rev3l*-deficient MEFs, the BL2 lines exhibited no statistically significant differences in cell doubling times, micronuclei formation or double-strand break formation as assessed by 53BP1 foci per cell. A modest sensitivity of 332 and 504 cells to UVC radiation and cisplatin was not rescued by complementation with *REV3L*. The limited sensitivity of 332 and 504 cells to a variety of DNA damaging agents has been noted [43–45]. Others have also reported no significant differences in spontaneous DNA breaks in 332 and 504 cells compared to wild-type BL2 cells [43]. In a study with wild-type BL2 cell extracts and extracts from the nominal *REV3L*-deficient cells [46], it was concluded that REV3L does not contribute to acetylaminofluorine-induced frameshift mutagenesis. This should probably be re-examined with a different *REV3L*-defective cell system. It is possible that the modest increased sensitivity of the BL2 subclones to UV radiation and cisplatin [43] might be due to inadvertent disruption of an unrelated gene by the targeting strategy, as may have occurred with BL2 cells deleted for pol ι [47–49]. Our data indicate that the 332 and 504 cell lines may not be truly (or only) *REV3L*-deficient, and are not well-suited for studies of REV3L function.

### Cooperation of pol η and pol ζ in conferring genome protection

The DT40 chicken cell line has been widely used to examine the consequences of DNA repair defects, because it is amenable to genetic manipulation by homologous recombination. Some characteristics of *Rev3l*-deficient DT40 cells are similar to *Rev3l*-deficient mouse cells, including elevated levels of spontaneous DNA breaks and sensitivity to DNA damaging agents. Intriguingly, it was reported that deletion of *polh* (pol η) could rescue the severe phenotypes of *Rev3l*-deficient DT40 cells [27]. This led to the model that the major defects in *Rev3l* –deficient cells are a consequence of a *polh*-dependent toxic intermediate. To test this model in mammalian cells, we investigated whether *Rev3l*^*-/-*^ *Polh*^-/-^ mice could be generated. We found that embryonic lethality of this double mutant was complete and similar in timing to *Rev3l*^*-/-*^ *Polh*^*+/+*^ mice. Moreover, *Rev3l*^*-/Δ*^ *Polh*^*-/-*^ MEFs showed levels of DNA breaks and cisplatin sensitivity analogous to that seen with *Rev3l* deletion in the presence of pol η. The *Rev3l*^-/lox^ *Polh*^-/-^ MEFs were more sensitive to cisplatin than the *Rev3l*^-/lox^ *Polh*^+/+^ MEFs, consistent with the cisplatin sensitivity of human *polh*-defective cells [35]. Notably, the pol η defective and pol η pol ζ double mutant MEFs had similar sensitivities to cisplatin. This epistatic interaction suggests that these two proteins act in the same pathway to mediate resistance to cisplatin. In fact both polymerases can cooperate to bypass a cisplatin-DNA adduct [24]. In summary, the severe phenotypes caused by *Rev3l* deletion cannot be rescued in murine cells by concurrent deletion of pol η. This is consistent with results found in the yeast *S*. *cerevisiae*, where a *Rev3 Rad30* (pol ζ pol η) mutant is more sensitive to ultraviolet radiation than a single *Rev3* mutant [50, 51]. Although the absence of pol η causes sensitivity to some DNA damaging agents, it is not specifically toxic in the absence of pol ζ. In the absence of pol ζ, it is possible that TLS does not occur at all, and that other modes of replication fork rescue are relied upon, which leads to a higher prevalence of DNA double-strand breaks [1]. The genetic interaction between pol η and pol ζ reported for chicken DT40 cells might reflect a peculiarity of that cell line. DT40 cells harbor mutations in *TP53*, and no *poli* gene has been found in the chicken genome. but it seems unlikely that either gene is relevant in this context. Previously reported *Tp53*^-/-^ *Rev3l*^-/-^ MEFs are also pol i deficient (an allele from the 129 ES cell background), and show major genome instability and DNA damage sensitivity [9]. *Polh poli* double mutant mice are apparently normal with no deficits in development. A *poli* defect does not exacerbate the UV radiation sensitivity of *polh*-defective mouse cells, indicating that pol ι does not have a significant backup function protecting against lethality in the absence of pol η [52].

### Implications for cancer therapy

Our results with the *Rev3l* knock-in polymerase mutant mouse are relevant to development of REV3L as a target for chemotherapy. Suppression of REV3L sensitizes cancer cells to cisplatin in mouse model systems, and can limit chemo-resistance [12, 53] because loss of pol ζ diminishes point mutagenesis [2–5]. These studies used siRNA knockdown of REV3L to demonstrate this effect, but future use of small molecule DNA polymerase inhibitors may be more clinically feasible. Until now it has not been known whether inhibition of the catalytic activity of REV3L mimics the cytotoxic effects of a knockdown of the entire gene. Our work demonstrates that loss of REV3L catalytic activity is equivalent, in the assays used here, to gene knockout. This validates and encourages strategies to directly inhibit pol ζ DNA polymerase activity.

## Materials and Methods

### Cell lines

*Rev3l*-deficient TAg-immortalized MEFs were derived as in Lange et al [8]. Briefly, MEFs were made from mouse embryos with the genotypes *mT/mG*^+/-^ *Rev3l*^*-/lox*^ or *mT/mG*^*+/-*^ *Rev3l*^*+/lox*^, where “lox” represents a functional allele flanked by loxP sites. The mT/mG transgene constitutively expresses RFP, until Cre recombinase activity removes the RFP and allows expression of GFP [34]. The strain background of the mice used to derive these alleles was mixed C57BL/6 and 129. We genotyped DNA polymerase iota (pol ι) in these cell lines, because 129 mice carry a mutant allele of pol ι [54]. All cell lines were heterozygous for this mutation, and so can be considered pol ι proficient. These cell lines were immortalized with SV40 large T-antigen, and then treated with adenovirus Cre (AdCre) to delete the floxed allele of *Rev3l* (and generate the knockout Δ allele). The cells were subcloned and selected for GFP positivity and for complete deletion of the floxed *Rev3l* allele. They were grown as in Lange et al [8], in an atmosphere containing 2% O_2_. The primary MEFs were also derived and cultured in 2% O_2_ as in Lange et al [8]. They were made from mouse embryos with the genotypes *mT/mG*^+/-^ *Rev3l*^M/lox^ or *mT/mG*^+/-^ *Rev3l*^+/lox^, as well as from *Rev3l*^-/lox^ *Polh*^-/-^ or *Rev3l*^-/lox^ *Polh*^+/+^ embryos. The loxP-flanked allele of the *Rev3l* gene was deleted using AdCre adfection, and the deletion efficiency was measured as described [8]. For all cell lines, cell number was counted at each passage, and was used to calculate population doublings and doubling time.

The BL2 parental cell line and subclones 332 and 504 [31] were kindly provided by Claude-Agnés Reynaud (Institut Gustave Roussy, Villejuif, France). Genomic DNA samples from the three cell lines were compared using short tandem repeat (STR) fingerprinting by the Cell Line Identification Core at MD Anderson. All yielded identical profiles of the 16 standard STR markers, confirming the relationship of the three cell lines.

### Expression of human *REV3L* in cell lines

The human *REV3L* full-length cDNA was acquired in the pUC19M1 vector from Zhigang Wang [55]. The following modifications were made to the *REV3L* cDNA: a C-terminal Flag tag was added and the 5’-UTR was eliminated and replaced with an optimized mammalian Kozak sequence. This cDNA was cloned into the pTSIGN vector, which contains an EF1α promoter and an internal ribosomal entry site (IRES) fused to a neomycin-eGFP reporter. The active site mutation (residues D2781A/D2783A in human *REV3L)* was introduced into this pTSIGN-REV3L vector using PCR primers containing the *REV3L* mutations, and then the mutated PCR fragment was ligated into the *REV3L*-vector, replacing the wild-type sequence. The full-length human *REV3L* gene was PCR amplified from the pTSIGN-REV3L and pTSIGN-REV3L-ASM vectors and was cloned into the pETDuet-1 vector (Novagen). The *REV3L* gene was removed from the *REV3L*-pETDuet-1 vectors using XhoI/NotI digestion, and the resulting fragments were inserted into the pOZN vector (contains a Flag-HA tag on the N-terminal side of the inserted gene [28]). For the pCDH vector, the XhoI/NotI fragment from the full-length *REV3L*-pETDuet-1 vector was inserted into the pCDH-EF1α-Flag-HA-MCS-IRES-Puro vector (System Biosciences). All vectors were completely sequenced to verify the integrity of the *REV3L* gene and the plasmid backbone. Full-length Flag-HA tagged REV3L can be expressed from this cDNA [19].

The pOZN-REV3L or pCDH-REV3L vectors were transfected into HEK-293T cells using lipofectamine 2000 (Life Technologies), together with the retroviral packaging vectors psPAX2 (plasmid 12260, Addgene) and pMD2.G (plasmid 12259, Addgene). 48 hr later, the media (containing pOZ or pCDH lentivirus) was collected. It was filtered, and polybrene was added to 4 μg/mL. This media was added to plates of immortalized MEFs (pOZ) or flasks of BL2 cells (pCDH). 48 hr later, the cells began selection for puromycin expression (pCDH, 10 day incubation), or for IL2R expression (pOZ). The latter required incubation of the infected cells with IL2R-antibody conjugated magnetic beads followed by washing of the beads (as in [56, 57]; IL2R antibody from Millipore, 05–170). This was repeated 5 times. The population was then sorted for single-cells, and clones were selected and verified. The cells were confirmed to contain both the N and C-terminal portions of the *REV3L* expression construct using the following PCR primers: NFwd: 5’ TAC ACA GTC CTG CTG ACC AC 3’, NRev: 5’ GAG GTA AGG AAA GAT GCC ATG TAG 3’, CFwd: 5’ ACC TAA CTC AGC ATG GCA TCT G 3’, CRev: 5’ CGG AAT TGA TCC GCT AGA G 3’ (at an annealing temperature of 50°C).

Expression of the recombinant human REV3L was confirmed using a human-specific Taqman assay (Life Technologies) at the exon 14–15 boundary: Ex14Fwd: 5’ CAC CTG GCC TTA GCC CAT TAT 3’, Ex15Rev: 5’ CTC TTC TAA GAG TGT CAG TAT TAC TTC CTT TC 3’ Probe: FAM-MGB-5’ CAA CAG AAC CAA AAA CA 3’. In order to compare the recombinant expression to that of endogenous mouse *Rev3l*, we designed a set of primers and a probe that would recognize both human and mouse *Rev3l*, and would not amplify any knockout transcript. The primers/probe were at the exon 26/27 boundary: Ex26Fwd: 5’ GTG AAT GAT ACC AAG AAA TGG GG 3’; Ex27Rev: 5’ GTG AAT GAT ACC AAG AAA TGG GG 3’; Probe: FAM-MGB-5’ TAC TGA CAG TAT GTT TGT 3’. An additional gene expression analysis was completed on the hREV3L-expressing BL2 cells in order to distinguish the endogenous REV3L transcript (which was expressed at approximately equal levels in the REV3L knockout and wild-type BL2 cells) from the exogenously expressed REV3L. We used primers and a probe that crossed the FLAG tag on the exogenous gene: FlagFwd: 5’–GTCTTTGTTTCGTTTTCTGTTCTG C– 3’; FlagRev: 5’–GCTTGTCATCGTCGTCCTTG– 3’; Probe: FAM-MGB-5’–GCT GTG ACC GGC GCC TAC TCT AG– 3’. Gene expression (with mouse or human GAPDH as an expression control) was measured on an Applied Biosystems 7900HT Fast Real-Time PCR System.

### Mice

#### Ethics statement

All animal work in this study was done according to The University of Texas, MD Anderson Cancer Center Institutional Animal Care and Use Committee guidelines, and approved by the MD Anderson Animal Care and Use Committee (IACUC).

#### Construction of the targeting vector

The targeting vector construction and the FlEx strategy [32] (Cre-dependent genetic switch approach) were designed and performed by genOway (Lyon, France). The *Rev3l* targeting vector was constructed from C57BL/6 mouse strain genomic DNA with a long (5.7 kb) homology arm upstream of exon 27, and a short (1.5 kb) homology arm downstream of exon 27. A D2773A/D2775A *Rev3l* mutant exon 27 and a Neo cassette (selection marker flanked by FRT sites for use in Flp-mediated excision) were inserted into intron 26. The targeting vector also incorporated a diphtheria toxin negative selection cassette. The action of Cre recombinase switched the allele to express mutant REV3L via the use of a combination of specifically oriented loxP and lox511 sites flanking the exons, as in Fig 4A.

#### Screening of *Rev3l*^D2773A/D2775A-Neo^ targeted ES cell clones

Linearized targeting vector was transfected into C57BL/6 ES cells (genOway, Lyon, France) according to genOway's electroporation procedures (i.e. 5 x 10^6^ ES cells with 40 μg of linearized plasmid, 260 V, 500 μF). Positive selection started 48 hr after electroporation in medium containing 200 μg/ml of G418 (150 μg/ml of active component, Life Technologies, Inc.). *Rev3l* resistant clones were isolated and amplified in 96-well plates. Duplicates of 96-well plates were made. The set of plates containing ES cell clones amplified on gelatin were genotyped by both PCR and Southern blot analysis.

For PCR analysis, one primer pair was designed to amplify sequences spanning the 3’ homology region. This primer pair was designed to specifically amplify the targeted locus:

Forward (Neo cassette): 5’-ATGCTCCAGACTGCCTTGGGAAAAG-3'

Reverse: 5'-CTGGGGTGCTACTGTTCTTGTTAGAGTGC-3'

A second PCR was designed to confirm the integration of the FlEx cassette (mutant exon 27 and *lox*P/lox511 sites):

Forward: 5’- GCCAAAGAGACATGCAGTGAGAAGAGTACC-3'

Reverse: 5'- TGAGTGGGCTTGCAGAAGTCAGCA-3'

PCR products were then sequenced in order to validate the presence of all FlEx elements. The targeted locus was confirmed by Southern blotting using internal and external probes for both 3’ and 5’ ends (Fig 3). Eight clones were identified as correctly targeted at the *Rev3l* locus.

#### Generation of mosaic mice and breeding scheme

Clones were microinjected into albino C57BL/6 blastocysts, and gave rise to male mosaics with a significant ES cell contribution (as determined by a black coat color). Mice were bred to C57BL/6 mice expressing the Flp recombinase to remove the Neo cassette (*Rev3l*^D2773A/D2775A-flox^ mice), which resulted in the inducible allele gene architecture. These mice were then crossed with CMV-Cre mice to remove the endogenous exon 27 and allow expression of the mutant exon 27 cassette, resulting in the constitutive ASM allele (*Rev3l*^D2773A/D2775A^ mice) (Fig 3A).

#### Genotyping of the *Rev3l*^D2773A/D2775A-flox^ inducible mouse line

The following genotyping primers were used to genotype the inducible *Rev3l*^D2773A/D2775A-flox^ allele:

Forward: 5’- GCCAAAGAGACATGCAGTGAGAAGAGTACC-3'

Reverse: 5'- TGAGTGGGCTTGCAGAAGTCAGCA-3'

No amplification is expected for the wild-type allele, 1680-bp for the *Rev3l*^D2773A/D2775A-flox^ allele, 3353-bp for the *Rev3l*^D2773A/D2775A-Neo^ allele. Animals were then validated by Southern blot analysis using a 3’ external probe: the wild-type allele gives rise to a 15 kb signal while the *Rev3l*^D2773A/D2775A-flox^ allele gives rise to a 10.4 kb signal, and the *Rev3l*^D2773A/D2775A-Neo^ allele is expected to give a signal at 11.5 kb.

#### Genotyping of the *Rev3l*^D2773A/D2775A^ constitutive mouse line

The following genotyping primers were used to genotype the induced *Rev3l*^D2773A/D2775A^ mice:

Forward: 5’- GCCAAAGAGACATGCAGTGAGAAGAGTACC-3'

Reverse: 5'- TGAGTGGGCTTGCAGAAGTCAGCA-3'

No amplification is expected for the wild-type allele, 1680-bp for the *Rev3l*^D2773A/D2775A-flox^ allele, 1131 bp for the *Rev3l*^D2773A/D2775A^ allele.

Animals were then validated by Southern blot analysis using a 3’ external probe: the wild-type allele gives rise to a 15 kb signal, the *Rev3l*^D2773A/D2775A^ allele gives rise to a 9.5 kb signal, and the *Rev3l*^D2773A/D2775A-Neo^ allele is expected to give a signal at 11.5 kb (Fig 3).

The presence of the mutant allele in the mice can be confirmed by PCR using the following primers: ASMWTFwd: 5’ TTG GGG CAT TGG TTT ACA GGT GGG 3’ and ASMWTRev: 5’ GCT GCT GAT ACT ACT ACT ACC ACC ACC ACT ACC 3’. Using these primers, the wild-type allele produces a 236 bp product, and the mutant allele a 345 bp product (at an annealing temperature of 65°C). Heterozygous mutant mice (which were maintained as pure C57BL/6 mice) were crossed in an attempt to observed homozygous mutant progeny. When this was unsuccessful, embryos were isolated at days 8.5–12.5 dpc. No viable embryos that were homozygous mutant were identified. To produce *Rev3l* mutant/floxed mice, *Rev3l*^+/M^ mice were crossed to mT/mG^+/+^, *Rev3l*^-/lox^ mice (the latter were on a C57BL6/N; 129 strain).

To investigate the phenotypes of animals and cell lines that were knockout for both *Rev3l* and DNA polymerase η, we obtained mice from P. Gearhart with the genotype *Rev3l*^-/lox^ *Polh*^-/-^ (*Rev3L* heterozygous and *Polh* knockout, as in [58]). These mice were in a mixed C57BL/6 and 129X1 background (formerly termed 129/SvJ) background and all had a pure white color. SNP analysis by the MD Anderson Genetics Services Core showed that the mice analyzed were homozygous for 129X1 alleles throughout the region of chromosome 7 flanking the albino Tyr locus. Because the 129Sv strain contains a nonsense mutation in pol ι [54] we genotyped pol ι in these *Rev3l*^-/lox^, *Polh*^-/-^ mice and determined that they harbored the *Poli*^+/+^ C57BL/6 allele (homozygous for wild-type pol ι). After crossing *Rev3l*^-/lox^ *Polh*^-/-^ mice, the resulting progeny were genotyped as in Lange et al [8], and Saribasak et al [58]. Embryos were also isolated at e9.5 and e10.5 dpc, but no viable *Rev3l*^-/-^, *Polh*^-/-^ embryos were identified. To study the phenotypes of *Rev3l* and pol η deficient cell lines, we crossed *Rev3l*^-/lox^, *Polh*^-/-^ mice and isolated MEFs with this genotype.

### DNA damage sensitivity

To test sensitivity to chemical DNA damaging agents, the immortalized MEFs or BL2 cells were plated into white 96-well plates (immortalized MEFs– 5,000 cells/well; BL2 cells– 10,000 cells/well). The following day, various concentrations of cisplatin (Sigma) or bleomycin (Sigma) were added to the wells, and the cells were incubated for 48 hr. Then the cells were lysed, a reagent was added that emits light in the presence of ATP (ATPLite One Step, Perkin Elmer), and luminescence was measured using a plate reader (Biotek Synergy II). The luminescence measurement was normalized to undamaged control. To test cisplatin sensitivity in *Rev3l*-deleting primary MEFs, 1 day after deleting the *Rev3l* floxed allele with AdCre, the cells were plated into white 96-well plates (10,000 cells/well). On day 3, cisplatin at various concentrations was added, and the cells were incubated for 5 days. Then ATP content was measured by luminescence, as above.

To test sensitivity of immortalized MEFs or BL2 cells to UVC radiation, 3 x 10^5^ cells were pelleted and resuspended in 300 μL of phosphate-buffered saline. Three 100 μL drops were placed into the middle of a plastic dish and 10 μL aliquots from each were plated into 100 μL of growth media in a white 96-well plate after 0, 2.5, 5, 7.5, 10, 15 or 20 J/m^2^ UVC radiation at a fluence of 0.4 J/m^2^ s^-1^. 48 hr after irradiation, ATP content was measured as above.

### Immunofluorescence

To measure the formation of DNA double-strand breaks, immortalized MEFs were plated in an 8-well chamber slide. The following day they were fixed and stained for DAPI, 53BP1 and γ-H2AX, as in Lange *et al* [8]. BL2 cells were applied to microscope slides using a Cytospin (Thermo Scientific), and then fixed and stained as with the MEFs. Immunofluorescence images were photographed through a Leica DMI6000B microscope. Micronuclei were counted based on small, separate DAPI foci associated with DAPI-stained nuclei. 53BP1 foci per cell were counted using the CellProfiler program 1 [59] with a threshold correction factor of 1.7. To measure DNA double-strand breaks in the primary MEFs, cells were plated into 8-well chamber slides 7 days after deletion of the *Rev3l* floxed allele using AdCre. 48 hr later they were fixed and stained for DAPI, 53BP1 and γ-H2AX as above. Photographs of the immunofluorescence were taken on the Leica microscope, and cells containing double-strand breaks were scored as those with 3 or more 53BP1 + γ-H2AX foci.

### Chromosomal analysis

Assessment of the Rev3L^-/Δ^ and Rev3L^+/Δ^ cell lines for sister chromatid exchanges (SCEs) was as described [57]. BrdU (10 μM) was added to growing TAg-immortalized MEFs for a period of two cell cycles, followed by a 4 hr incubation with 0.02 μg/mL colcemid. The cells were then harvested and incubated with hypotonic solution (0.075 M KCl) for 10 min at 37°C. Then 50 μL of fresh fixative solution (3:1 methanol:acetic acid) was added and the cells were pelleted at 1000 rpm for 10 min. After aspiration of the supernatant, 5 mL of fresh 4°C Carnoy’s fixative (6:3:1 ethanol: chloroform: glacial acetic acid) was added dropwise to the pellet and the cells were incubated at 4°C for 30 min followed by centrifugation for 10 min at 1000 rpm at 4°C. The supernatant was aspirated, and this process was repeated. 1 mL of Carnoy’s fixative was added to the final cell pellet and the cells were dropped onto clean microscope slides in a humid environment to favor chromosome spreading. The slides were stained for 45 min in 0.5X SSC buffer containing 2 μg/mL Hoechst 33258 for 45 min, and then were washed twice in SSC buffer for 5 min each. The slides were then immersed in 0.5X SSC buffer and exposed to UVA light (350 nm wavelength, 15 W) at a distance of 10 cm for 1 hr. Then the slides were incubated for 1 hr in fresh 0.5X SSC buffer at 60°C, and were stained for 15 min with 3% Giemsa dye in Sorenson’s buffer (Sigma, diluted 1:15 in 0.025 M KH_2_PO_4_ pH 6.8). The chromosome spreads were viewed at 600X magnification under oil. Thirty to thirty-five chromosome spreads were counted for each genotype, and both total chromosome number and number of SCEs was assessed.

### Statistical analysis

Analysis of cells with associated micronuclei or > 2 53BP1 + γ-H2AX foci was done using one-way ANOVA with Tukey’s multiple comparisons test (P < 0.05). Statistical analysis of cell survival after cisplatin or UVC treatment, or of cell growth, was done using linear regression analysis, and the lines were compared based on equality of slope and intercepts.

## Supporting Information

S1 Fig Three regions of highest identity/similarity between REV3L and the KIAA2022 gene. Mammalian REV3L is about twice as long as *S*. *cerevisiae* Rev3, owing largely to a central domain of 1500 amino acids. A Blast search using the central domain detects similarity to the *KIAA2022* gene on the human X chromosome, as we noted previously [60]. The KIAA2022 protein is predicted to have 1516 amino acids, largely encoded by exon 3 of the four exon gene [61]. KIAA2022 is disrupted in a family with X-linked mental retardation [61]. Most of the central domain of mammalian REV3L is encoded by a single large exon (Exon 14, 4162 bp in human *REV3L*), encoding 1386 amino acids. A Clustal X alignment, using default parameters, was generated by aligning this region of the indicated REV3L orthologs with the 1458 residue human KIAA2022 exon 3-encoded region from the same species. Most homology between the *KIAA2022* and *REV3L* gene products is confined to a region of about 250 amino acids, with the three regions of highest similarity shown here. The third region includes a proposed nuclear localization signal [62]. Part of KIAA2022 appears to have been retrotransposed to the genome of a multicellular eukaryote ancestor of the *REV3L* gene. This is an example of the frequent gene traffic between the mammalian X chromosome and autosomes observed during evolution [63].
(TIFF)

S2 Fig Lack of *REV3L* mutant phenotypes in human BL2 cell lines. Two reported human REV3L knockout lines designated 332 and 504 were derived from the Burkitt lymphoma cell line BL2 [31]. We expressed human *REV3L* in these cell lines using a pCDH vector system with puromycin as a selectable marker (B-cells express IL2R, preventing the use of pOZ vectors [64]). We have successfully expressed *REV3L* in human cells using pCDH [19]. **(A)** Expression of recombinant Flag-tagged empty vector in BL2 WT cells or recombinant Flag-tagged REV3L in 332 cells. The 332 and 504 cells still expressed detectable levels of endogenous *REV3L* cDNA, but expression of exogenous recombinant *REV3L* could be achieved above the endogenous level. Expression of exogenous REV3L cDNA was confirmed using primers specific to that cDNA. **(B)** Doubling time (in hr) of BL2 WT, REV3L-deficient 332 and 504 cells, and 332 cells expressing exogenous REV3L. Expression of wild type *REV3L* cDNA did not alter the doubling time of these cells. **(C)** Survival of the same cell lines as in (B) as measured by ATP concentration 48 hr after addition of cisplatin. The 332 and 504 cell lines were not more sensitive to cisplatin than the parental cells. **(D)** Survival of the cell lines as measured by ATP concentration 48 hr after ultraviolet C (UVC) radiation. Both lines appeared moderately UVC radiation-sensitive, but *REV3L* re-expression did not rescue this sensitivity. **(E)** Quantification of percent of BL2 cell nuclei with associated micronuclei. **(F)** Quantification of cells with fewer than 3, 3 to 5, 6 to 10 or greater than ten 53BP1 foci (as measured using CellProfiler). There was no significant elevation in the spontaneous incidence of DNA double-strand breaks in 332 or 504 cells (compare S2E and S2F Fig with Fig 2C and 2D). Data represent mean ± SEM.
(TIFF)

S3 Fig Analysis of the targeting strategy used for BL2 cells. The major transcript variants of human REV3L are shown. Transcript variant 1 (NM_002912) encodes DNA polymerase ζ catalytic subunit isoform a, with 33 exons translating to 3130 aa. Transcript variant 2 (NM_001286431) encodes DNA polymerase ζ catalytic subunit isoform b, with 35 exons translating to 3052 aa. The two additional exons in transcript variant 2 are indicated (yellow). A short upstream ORF is also present in transcript variant 2. Gueranger *et al*., who made the 332 and 504 cell lines, designed a targeting strategy with the intention of removing the exon encoding the initiating ATG codon for REV3L, and a downstream exon [31]. In retrospect, this targeting was designed for transcript variant 2, and affects exons 3 and 4 of the major *REV3L* transcript variant 1. The bottom part of the figure shows the location of the primer pair given in [31] to assess targeting of the *neo* marker intended to disrupt exon 5. The primer 3'-KO-zeta-2 for this analysis is located 100 kb away from exons 5, a distance not compatible with PCR genotyping. We note also that both primers used to test absence of *Rev3l* mRNA by Gueranger *et al*. are located in the deleted region, and therefore no PCR product would be generated in cells even if *REV3L* mRNA were present.
(TIFF)
