# Supplementary figures and images for: The Polymerase Activity of Mammalian DNA Pol ζ Is Specifically Required for Cell and Embryonic Viability
Source: PLoS Genet. 2016 Jan 4;12(1):e1005759. doi: 10.1371/journal.pgen.1005759 (PMC4699697; doi:10.1371/journal.pgen.1005759)

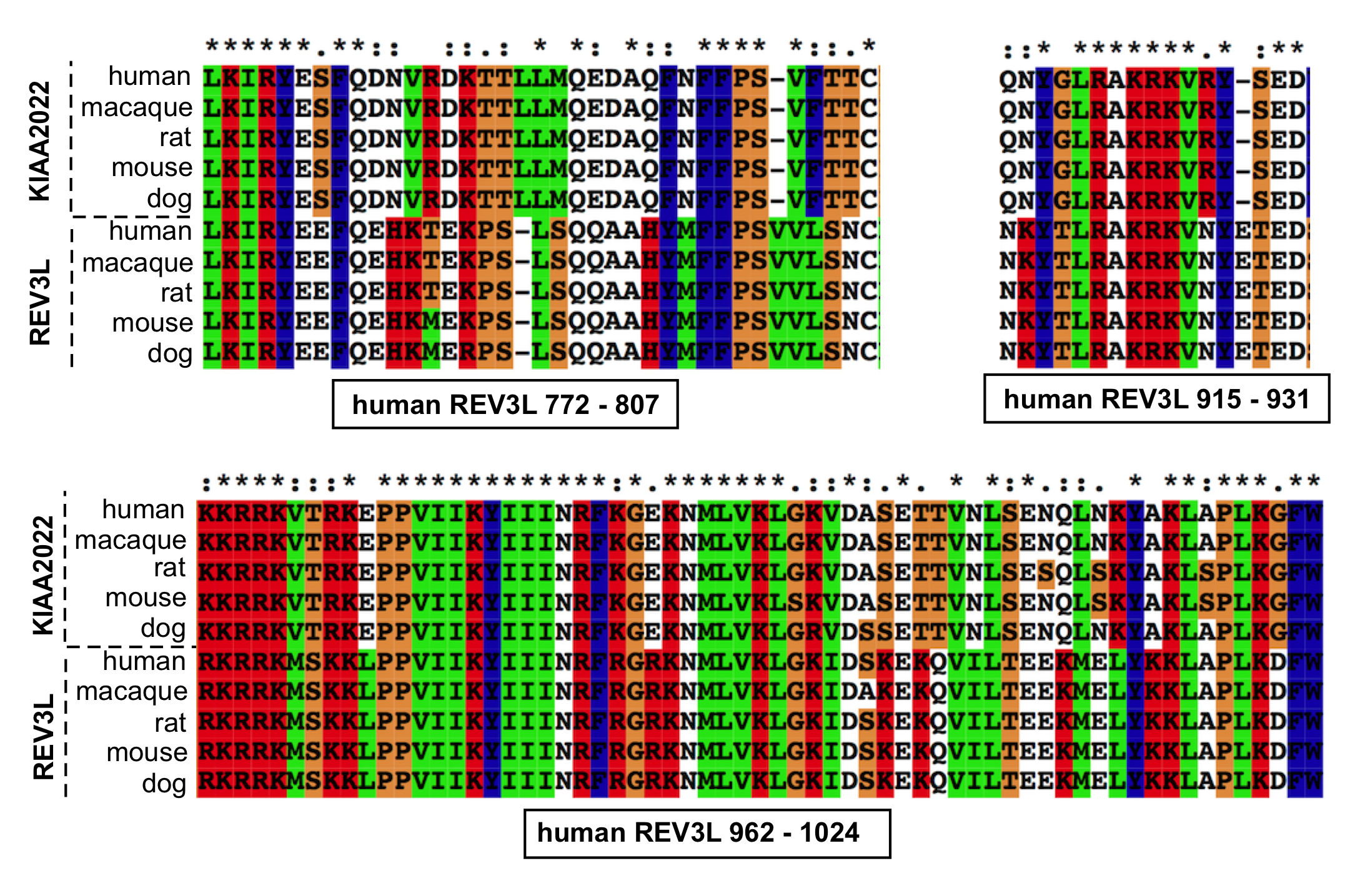

Supplement: S1 Fig — Mammalian REV3L is about twice as long as S. cerevisiae Rev3, owing largely to a central domain of 1500 amino acids. A Blast search using the central domain detects similarity to the KIAA2022 gene on the human X chromosome, as we noted previously [60]. The KIAA2022 protein is predicted to have 1516 amino acids, largely encoded by exon 3 of the four exon gene [61]. KIAA2022 is disrupted in a family with X-linked mental retardation [61]. Most of the central domain of mammalian REV3L is encoded by a single large exon (Exon 14, 4162 bp in human REV3L), encoding 1386 amino acids. A Clustal X alignment, using default parameters, was generated by aligning this region of the indicated REV3L orthologs with the 1458 residue human KIAA2022 exon 3-encoded region from the same species. Most homology between the KIAA2022 and REV3L gene products is confined to a region of about 250 amino acids, with the three regions of highest similarity shown here. The third region includes a proposed nuclear localization signal [62]. Part of KIAA2022 appears to have been retrotransposed to the genome of a multicellular eukaryote ancestor of the REV3L gene. This is an example of the frequent gene traffic between the mammalian X chromosome and autosomes observed during evolution [63]. (TIFF) [file pgen.1005759.s001.tiff]

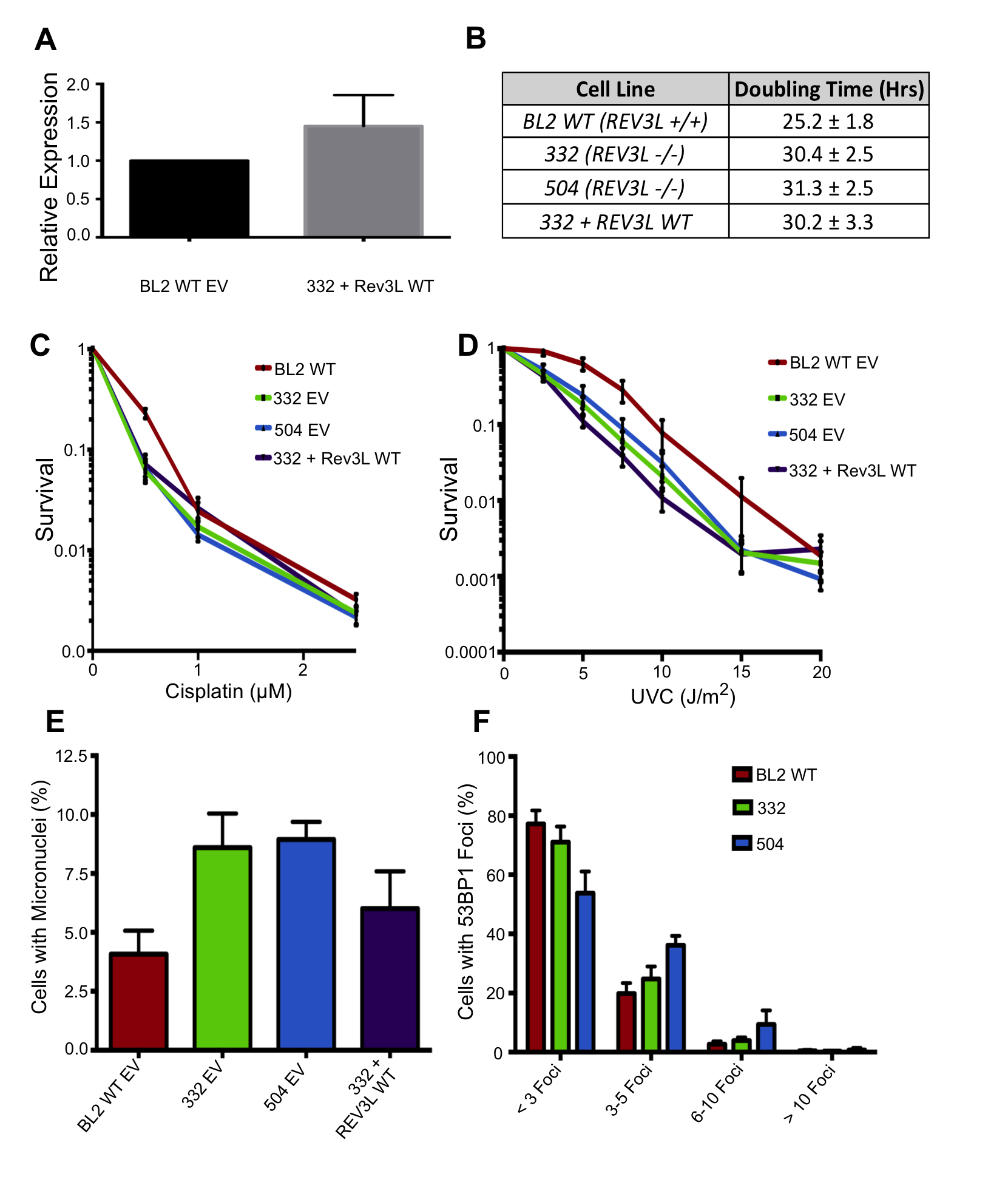

Supplement: S2 Fig — Two reported human REV3L knockout lines designated 332 and 504 were derived from the Burkitt lymphoma cell line BL2 [31]. We expressed human REV3L in these cell lines using a pCDH vector system with puromycin as a selectable marker (B-cells express IL2R, preventing the use of pOZ vectors [64]). We have successfully expressed REV3L in human cells using pCDH [19]. (A) Expression of recombinant Flag-tagged empty vector in BL2 WT cells or recombinant Flag-tagged REV3L in 332 cells. The 332 and 504 cells still expressed detectable levels of endogenous REV3L cDNA, but expression of exogenous recombinant REV3L could be achieved above the endogenous level. Expression of exogenous REV3L cDNA was confirmed using primers specific to that cDNA. (B) Doubling time (in hr) of BL2 WT, REV3L-deficient 332 and 504 cells, and 332 cells expressing exogenous REV3L. Expression of wild type REV3L cDNA did not alter the doubling time of these cells. (C) Survival of the same cell lines as in (B) as measured by ATP concentration 48 hr after addition of cisplatin. The 332 and 504 cell lines were not more sensitive to cisplatin than the parental cells. (D) Survival of the cell lines as measured by ATP concentration 48 hr after ultraviolet C (UVC) radiation. Both lines appeared moderately UVC radiation-sensitive, but REV3L re-expression did not rescue this sensitivity. (E) Quantification of percent of BL2 cell nuclei with associated micronuclei. (F) Quantification of cells with fewer than 3, 3 to 5, 6 to 10 or greater than ten 53BP1 foci (as measured using CellProfiler). There was no significant elevation in the spontaneous incidence of DNA double-strand breaks in 332 or 504 cells (compare S2E and S2F Fig with Fig 2C and 2D). Data represent mean ± SEM. (TIFF) [file pgen.1005759.s002.tiff]

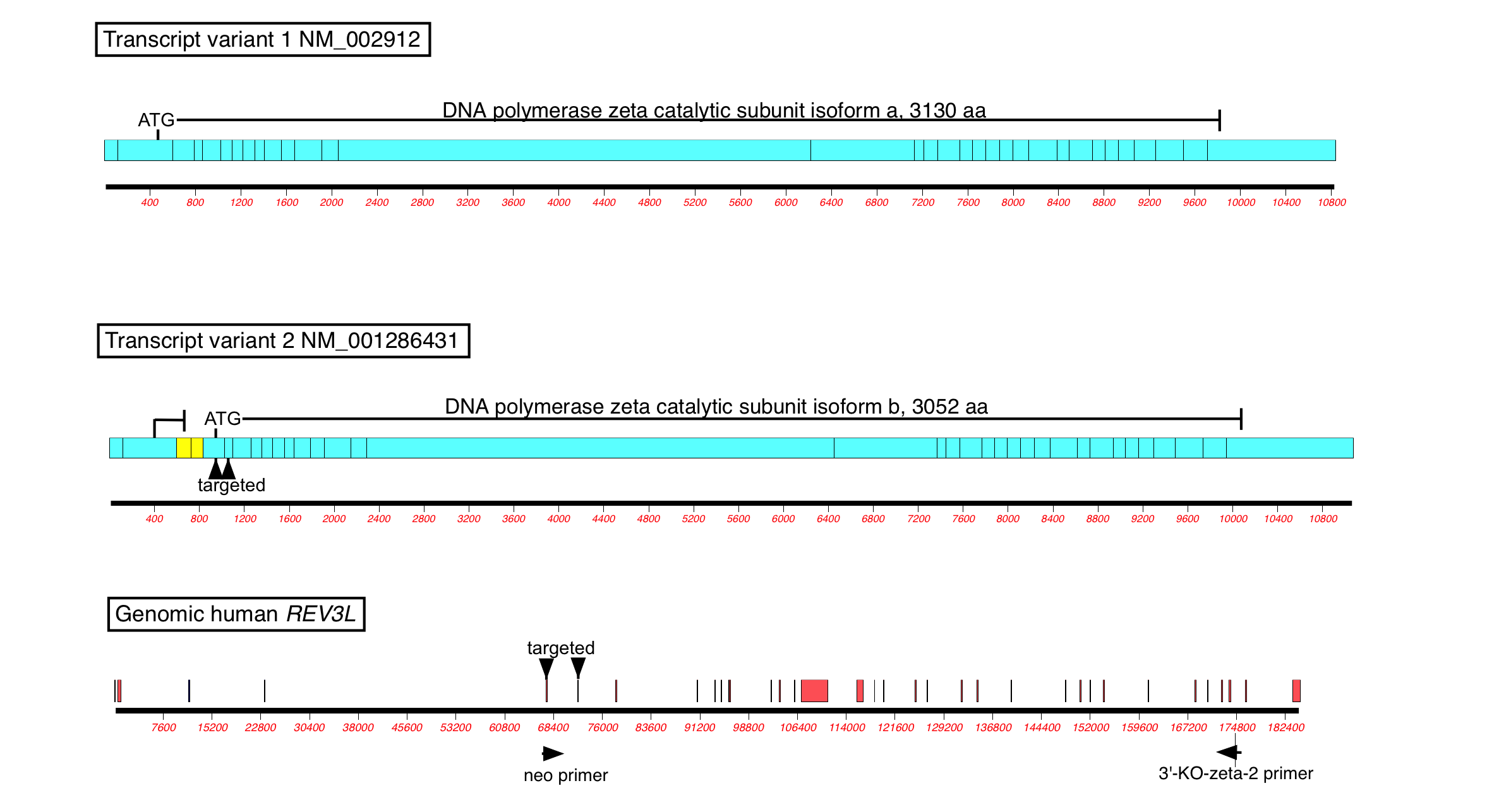

Supplement: S3 Fig — The major transcript variants of human REV3L are shown. Transcript variant 1 (NM_002912) encodes DNA polymerase ζ catalytic subunit isoform a, with 33 exons translating to 3130 aa. Transcript variant 2 (NM_001286431) encodes DNA polymerase ζ catalytic subunit isoform b, with 35 exons translating to 3052 aa. The two additional exons in transcript variant 2 are indicated (yellow). A short upstream ORF is also present in transcript variant 2. Gueranger et al., who made the 332 and 504 cell lines, designed a targeting strategy with the intention of removing the exon encoding the initiating ATG codon for REV3L, and a downstream exon [31]. In retrospect, this targeting was designed for transcript variant 2, and affects exons 3 and 4 of the major REV3L transcript variant 1. The bottom part of the figure shows the location of the primer pair given in [31] to assess targeting of the neo marker intended to disrupt exon 5. The primer 3'-KO-zeta-2 for this analysis is located 100 kb away from exons 5, a distance not compatible with PCR genotyping. We note also that both primers used to test absence of Rev3l mRNA by Gueranger et al. are located in the deleted region, and therefore no PCR product would be generated in cells even if REV3L mRNA were present. (TIFF) [file pgen.1005759.s003.tiff]
